# High-throughput analysis of the human thymic Vδ1^+^ T cell receptor repertoire

**DOI:** 10.1038/s41597-019-0118-2

**Published:** 2019-07-04

**Authors:** Biagio Di Lorenzo, Sarina Ravens, Bruno Silva-Santos

**Affiliations:** 1 Instituto de Medicina Molecular, Faculdade de Medicina, Universidade de Lisboa, Lisbon, Portugal; 2 Instituto Superior Técnico, Universidade de Lisboa, Lisbon, Portugal; 3 Institute of Immunology, Hannover Medical School, Hannover, Germany

**Keywords:** Immunogenetics, Next-generation sequencing, Gammadelta T cells

## Abstract

γδ T cells are a relatively rare subset of lymphocytes in the human peripheral blood, but they play important roles at the interface between the innate and the adaptive immune systems. The γδ T cell lineage is characterized by a signature γδ T cell receptor (γδTCR) that displays extensive sequence variability originated by DNA rearrangement of the corresponding V(D)J loci. Human γδ T cells comprise Vγ9Vδ2 T cells, the major subset in the peripheral blood; and Vδ1^+^ T cells, the predominant subpopulation in the post-natal thymus and in peripheral tissues. While less studied, Vδ1^+^ T cells recently gathered significant attention due to their anti-cancer and anti-viral activities. In this study we applied next-generation sequencing (NGS) to analyse the γδTCR repertoire of highly (FACS-)purified Vδ1^+^ T cells from human thymic biopsies. Our analysis reveals unsuspected aspects of thymically rearranged and expressed (at the mRNA level) *TRG* and *TRD* genes, thus constituting a data resource that qualifies previous conclusions on the TCR repertoire of γδ T cells developing in the human thymus.

## Abstract (summary)

**Table:** 

Design Type(s)	transcription profiling design • cellular data analysis objective
Measurement Type(s)	gamma delta T-lymphocyte
Technology Type(s)	RNA sequencing
Factor Type(s)	sex • gene
Sample Characteristic(s)	Homo sapiens • thymus

Machine-accessible metadata file describing the reported data (ISA-Tab format)

## Background & Summary

γδ T cells constitute a small (~5–10%) but unique subpopulation of T cells. Because of their features, including antigen recognition through a somatically rearranged T cell receptor (TCR) and several NK cell receptors^1^, cytokine production and immunoregulation^2^, they act as a bridge between innate and adaptive immunity, rapidly responding to infected or transformed cells in a major histocompatibility complex (MHC)-independent manner^1^.

Contrary to their αβ counterparts, there is little evidence supporting the hypothesis of human γδ T cells being positively and/or negatively selected in the thymus. Indeed, post-natal human γδ T cells seem to only complete their maturation program in the periphery, especially upon infection challenge that triggers clonal expansion^3–7^. Thus, the goal of post-natal thymic development seems to be the generation of a highly diverse, naïve and immature human γδ T cell repertoire. As such, the main event for developing γδ T cells is the generation of a functional TCR through the rearrangement of the variable (V), diversity (D) and joining (J) segments and subsequent pairing of the rearranged γ- and δ-chains. These events alone could lead to the establishment of a large number of diverse antigen receptors, but the addition and/or subtraction of non-templated (N) and palindromic (P) nucleotides at the gene segment junctions contribute substantially to increasing diversity, providing nearly limitless potential to the TCRγδ repertoire^8^.

There are several subsets of human γδ T cells, identified by the combination of rearranged TCRγ- and δ-chains. 14 TRGV genes, of which only 6 are functional (Vγ2, Vγ3, Vγ4, Vγ5, Vγ8 and Vγ9), can rearrange with 5 TRGJ (JP1, JP, J1, JP2 and J2) genes, whereas 7 TRDV genes (Vδ1, Vδ2, Vδ3 and Vα14/Vδ4, Vα23/Vδ6, Vα29/Vδ5, Vα36/Vδ7, Vα38-2/Vδ8) can rearrange with three TRDD (D1, D2 and D3) and with four TRDJ genes (J1, J4, J2 and J3)^9^. Although the thymus can generate all the possible combinations of TRG and TRD genes, the major γδ T cell population in peripheral blood (PB) expresses a TCR composed by Vγ9 recombined with JP and paired with Vδ2^10^. This specifically rearranged Vγ9Vδ_2_ T cell subset is mostly produced during foetal life but still constitutes the major γδ T cell subset in adults. Antigen-driven stimulation in the periphery underlies a strong and specific expansion of this subset after birth and during the lifespan of each individual^11^. Indeed, Vγ9Vδ2 T cells can rapidly recognize, in a TCR-dependent manner, cellular dysregulation resulting from infection or malignant transformation^1,10^.

Vδ1^+^ γδ T cells are the second most abundant subset in the human PB but the predominant γδ T cell subset in the post-natal thymus and in peripheral tissues (such as the intestine or the liver), where Vδ1 is mainly paired to Vγ8 or Vγ9 chains. These γδ T cells play an important role during viral infections, especially CMV^3,4^, and tumour progression^12^. Indeed, a subpopulation of Vδ1^+^ T cells recognizes nonpolymorphic MHC-like (class Ib) proteins presenting lipids, such as CD1 proteins, in a similar way to other unconventional T cells like NKT or MAIT cells^1,13–15^.

Thanks to recent technical advances in the comprehensive analysis of TCR repertoires using next-generation sequencing (NGS) approaches, it has become more accessible to understand the dynamics of T cell development and homeostasis^5,16^, and T cell expansion in response to infections^4,17^ or tumours^18^. In this context, the purpose of this study is to characterize the Vδ1^+^ TCR repertoire in the thymus of young children, providing a detailed description of transcribed (mRNA) V(D)J recombination products in highly (FACS-)purified thymic Vδ1^+^ T cells. This allowed us to reveal unsuspected aspects of the rearranged and expressed *TRG* and *TRD* repertoires of this cell population with the striking presence of a big fraction (~20%) of Vδ2 sequences in the thymic *TRD* repertoire of all 8 donors. While a small fraction of Vδ2 sequences, like that found in the PB Vδ1^+^ T cell pool, could be due to contamination with Vδ2^+^ cells, this cannot explain the large fraction of Vδ2 sequences in the highly FACS-purified thymic CD3^+^ Vδ1^+^ Vδ2^−^ samples.

Moreover, PB and cord blood Vδ1^+^ T cells were described to display private *TRD* repertoires, in contraposition to the respective *TRG* repertoire that showed a fraction of common (shared among individuals) sequences^4,6^. Here, we show that is also the case for purified Vδ1^+^ thymocytes, albeit these shared TRG clonotypes consist of TRGJ1 gene segments. This raises the interesting question whether the public TRG clonotypes of Vδ1^+^ T cells are selected (upon ligand encounter) in the thymus; or simply the output of a favoured TCR rearrangement. This, in fact, is an open question also on the Vγ9-JPVδ2 rearrangement that is prevalent in foetal life^10^.

In sum, this study constitutes a resource providing new data and qualifying previous conclusions on the TCR repertoire of human thymic γδ T cells^16^. Critically, the unexpected presence of a large fraction of Vδ2 sequences in Vδ1^+^ thymocytes strongly advocates for the use of highly purified cell populations, ideally complemented by single-cell validation experiments, to avoid misinterpretations of NGS data in future studies.

## Methods

### Sample collection and preparation

Thymic specimens were routinely collected during paediatric corrective cardiac surgery, after obtaining written informed consent. The study was approved by the Ethics Board of the Faculdade de Medicina da Universidade de Lisboa. Buffy coats from healthy volunteers were obtained under the agreement (15.12.2003) between Instituto de Medicina Molecular – João Lobo Antunes and Instituto Português do Sangue e da Transplantação approved by the local ethical committee (Centro de Ética do Centro Hospitalar Lisboa Norte - Hospital de Santa Maria). Thymic samples (from 5 days old to 15 months old children) were processed by tissue dispersion and Histopaque-1077 (Sigma-Aldrich) density gradient. Peripheral blood was collected from buffy coat cells, diluted with 1 volume of PBS (Invitrogen Life Technologies), and separated on a Histopaque-1077 density gradient. γδ T cells were first isolated by magnetic cell sorting (MACS) using a negative selection strategy (αβ depletion kit by Miltenyi Biotech); and then stained with anti-TCRVδ1 (clone TS8.2), anti-TCRVδ2 (clone B6), anti-CD3 (clone HIT3a) and anti-TCRαβ (clone IP26) mAbs; and FACS-sorted (CD3^+^ TCRVδ1^+^) to >98% purity in FACS Aria III (BD Biosciences). Flow cytometry acquired data were analysed with FlowJo X software (Tree Star).

### TRG and TRD amplicon generation, library preparation and NGS

FACS-sorted cells were subjected to RNA extraction using the RNAeasy mini kit (Qiagen). Next, 12 µl mRNA of each sample was reverse transcribed with the Superscript III enzyme kit (Invitrogen) according to the manufacturers protocol. Next, CDR3 TRG and TRD sequencing amplicons were generated as described previously^4^ using 7 µl cDNA template. In detail, primers were designed for all functional variable (V) and constant (C) gene segments of the human TRG and TRD locus having following sequences (5′–3′) h*TRD*V1: TCAAGAAAGCAGCGAAATCC; h*TRD*V2: ATTGCAAAGAACCTGGCTGT; h*TRD*V3: CGGTTTTCTGTGAAACACATTC; h*TRD*V5/29: ACAAAAGTGCCAAGCACCTC; h*TRD*C1: GACAAAAACGGATGGTTTGG; h*TRG*V (2,3,4,5,8): ACCTACACCAGGAGGGGAAG; h*TRG*V9: TCGAGAGAGACCTGGTGAAGT; h*TRG*C (1,2): GGGGAAACATCTGCATCAAG. Moreover, Illumina adaptor sequences (GTCTCGTGGGCTCGGAGATGTGTATAAGAGACAG and TCGTCGGCAGCGTCAGATGTGTATAAGAGACAG) were added as overhangs All forward primers for either TRG or TRD were combined in equal concentrations. PCR conditions are as following 1 × PCR buffer (without MgCl_2_; Invitrogen), 1.5 mM MgCl_2_, 10 mM dNTPs, 0.5 μM forward primer mix, 0.5 μM reverse primer and 0.04 units recombinant Taq polymerase (Invitrogen). Cycling conditions were 3 min at 95 °C; 30 s at 95 °C, 30 s at 63 °C and 30 s 72 at °C, for 5 cycles; 30 s at 95 °C and 35 s 72 at °C, for 20–25 cycles; and 4 min at 72 °C. PCR samples were run for gel electrophoresis on a 1%-Agarose-Gel, PCR amplicons (350 bps for TRG and 250 bps) were excised and gel-purified according to the QIAquick gel extraction kit (Qiagen). The multiplex-based CDR3 amplicon generation strategy has been previously validated by the 5′RACE-based CDR3 amplicon generation methods^4^.

For Illumina Miseq analysis, PCR amplicons were further subjected to an index PCR. In that case, individual 10 µl purified PCR amplicons were combined with 10 µl Advantage II PCR buffer (Clontech), 5 µl 10 mM dNTP (Clontech), 5 µl N50X and 5 µl N70X index primer (Nextera Index Kit, Illumina), 1 µl Advantage II polymerase (Clontech) and 30 µl dH_2_O for eight additional PCR cycles. PCR products were purified using the Agencourt AMPure XP PCR purification kit (Beckman Coulter) and eluted in 100 µl dH^2^O. After quantifying DNA concentrations with the QuantIT PicoGreen Assay (Invitrogen) PCR amplicons were pooled in equal concentration to generate a 4 nM library pool. According to the Illumina Denature and Dilute Library guidelines the library was diluted to 10 pM, while 20% PhIX was added as a control. The library was subjected to Illumina MiSeq analysis (paired-end sequencing) using following parameters: Amplicon chemistry; dual index; read 1: 250 bps; read 2: 250 bps; FASTQ only; use adaptor trimming.

### Data processing and analysis

*TRG* and *TRD* FASTQ sequences were subjected to quality controls through FASTQC (http://www.bioinformatics.babraham.ac.uk/projects/fastqc), allowing only very good and reasonable quality scores. Next, paired-end *TRG* sequences were joined at their overlapping ends using FASTQ-join of the command-line tool ea-utils (https://expressionanalysis.github.io/ea-utils/) with default parameters, while *TRD* sequences were fully covered from both ends and were processed directly for annotation. All obtained *TRG* sequences (ranging from the gene-specific primers targeting the functional V-regions between the CDR1 and CDR2 regions until the 5′end of the C-region) and *TRD* sequences (ranging from the V-region specific primers binding 3′ of the CDR2 regions until 5′ of the C-region) were annotated using IMGT/HighV-Quest^19^.

All bioinformatics downstream analysis was conducted in the Ubuntu 16.04 OS using in-house bash and R scripts (version 3.4.4). In brief, only productive amino acid sequences were taken into consideration. Next, reads with unambiguous V-gene segment annotation were accepted for further analysis^20,21^, by selecting only productive sequences with a clearly assigned V-region. All *TRG* and *TRD* clonotypes were identified based on identical CDR3 region sequences. Clonotype counts were further used to retrieve an estimation of the diversity of the transcribed repertoires. VDJtools^22^ (version 1.1.1) and tcR^23^ (version 2.2.3) packages were used for the post analysis of the T cell receptor repertoires that included clonotype counts, their abundance and CDR3 length distribution measurements and representation, Treemap (https://CRAN.R-project.org/package=treemap) was used for the graphical representations of the repertoires and Vegan (https://CRAN.R-project.org/package=vegan) for the Shannon diversity index estimation.

Statistical analysis was performed using GraphPad Prism software. All data expressed as mean +/− SEM and the comparisons of two groups was made using the Mann-Whitney test.

## Data Records

FCS files of unsorted and FACS-sorted Vδ1^+^ and Vδ2^+^ thymocytes are available and deposited at flowrepository.org^24^. Two *fastq* files (Read 1 and Read 2) have been generated at the end of the sequencing and deposited in the SRA database. *Fastq* files of thymic TRG and TRD sequences are available and deposited at NCBI Sequence Read Archive with the following accession IDs: SRR7993359, SRR7993360, SRR7993357, SRR7993358, SRR7993355, SRR7993356, SRR7993353, SRR7993354, SRR7993361, SRR7993362, SRR7993351, SRR7993352, SRR7993349, SRR7993350, SRR7993347, SRR7993348^25^. Data are also available at figshare^26^. Blood-derived Vδ1^+^ TRG and TRD sequences used as control are available at NCBI Sequence Read Archive with the following experiment IDs: SRX4717064^27^, SRX4717060^28^, SRX4717069^29^, SRX4717052^30^, SRX4717078^31^, SRX4717057^32^. These experiments are part of a larger project, which is under the project accession SRP162140^33^. Sample accession numbers with related BioProjects^25,27–32^, their provenance and the experimental manipulation performed are summarized in Table 1.

**Table 1:** Sample and BioProject accession numbers and performed experimental manipulation.

Subjects	Protocol 1	Protocol 2	Protocol 3	Protocol 4	Protocol 5	Data	Bioproject accession
Thy01_TRG	Tissue dispersion	Density gradient cell separation	FACS-Sorting	RNA extraction	RNA-Seq	SRR7993359	PRJNA495594
Thy02_TRG	Tissue dispersion	Density gradient cell separation	FACS-Sorting	RNA extraction	RNA-Seq	SRR7993360	PRJNA495594
Thy03_TRG	Tissue dispersion	Density gradient cell separation	FACS-Sorting	RNA extraction	RNA-Seq	SRR7993357	PRJNA495594
Thy04_TRG	Tissue dispersion	Density gradient cell separation	FACS-Sorting	RNA extraction	RNA-Seq	SRR7993358	PRJNA495594
Thy05_TRG	Tissue dispersion	Density gradient cell separation	FACS-Sorting	RNA extraction	RNA-Seq	SRR7993355	PRJNA495594
Thy06_TRG	Tissue dispersion	Density gradient cell separation	FACS-Sorting	RNA extraction	RNA-Seq	SRR7993356	PRJNA495594
Thy07_TRG	Tissue dispersion	Density gradient cell separation	FACS-Sorting	RNA extraction	RNA-Seq	SRR7993353	PRJNA495594
Thy08_TRG	Tissue dispersion	Density gradient cell separation	FACS-Sorting	RNA extraction	RNA-Seq	SRR7993354	PRJNA495594
Thy01_TRD	Tissue dispersion	Density gradient cell separation	FACS-Sorting	RNA extraction	RNA-Seq	SRR7993361	PRJNA495594
Thy02_TRD	Tissue dispersion	Density gradient cell separation	FACS-Sorting	RNA extraction	RNA-Seq	SRR7993362	PRJNA495594
Thy03_TRD	Tissue dispersion	Density gradient cell separation	FACS-Sorting	RNA extraction	RNA-Seq	SRR7993351	PRJNA495594
Thy04_TRD	Tissue dispersion	Density gradient cell separation	FACS-Sorting	RNA extraction	RNA-Seq	SRR7993352	PRJNA495594
Thy05_TRD	Tissue dispersion	Density gradient cell separation	FACS-Sorting	RNA extraction	RNA-Seq	SRR7993349	PRJNA495594
Thy06_TRD	Tissue dispersion	Density gradient cell separation	FACS-Sorting	RNA extraction	RNA-Seq	SRR7993350	PRJNA495594
Thy07_TRD	Tissue dispersion	Density gradient cell separation	FACS-Sorting	RNA extraction	RNA-Seq	SRR7993347	PRJNA495594
Thy08_TRD	Tissue dispersion	Density gradient cell separation	FACS-Sorting	RNA extraction	RNA-Seq	SRR7993348	PRJNA495594
HD2_PB_gamma	—	Density gradient cell separation	FACS-Sorting	RNA extraction	RNA-Seq	SRR7878381	PRJNA491919
HD2_PB_delta	—	Density gradient cell separation	FACS-Sorting	RNA extraction	RNA-Seq	SRR7878385	PRJNA491919
HD5_PB_gamma	—	Density gradient cell separation	FACS-Sorting	RNA extraction	RNA-Seq	SRR7878376	PRJNA491919
HD6_PB_gamma	—	Density gradient cell separation	FACS-Sorting	RNA extraction	RNA-Seq	SRR7878367	PRJNA491919
HD5_PB_delta	—	Density gradient cell separation	FACS-Sorting	RNA extraction	RNA-Seq	SRR7878393	PRJNA491919
HD6_PB_delta	—	Density gradient cell separation	FACS-Sorting	RNA extraction	RNA-Seq	SRR7878388	PRJNA491919

## Technical Validation

**Fig. 1:**
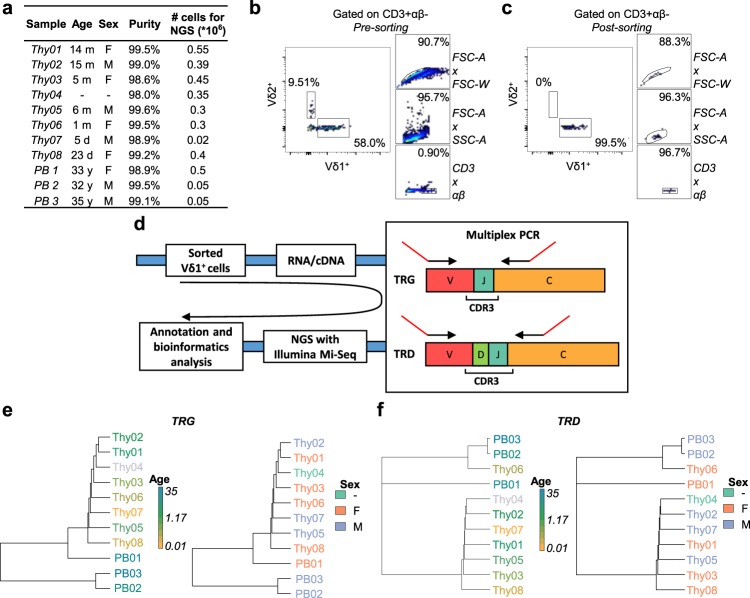
Summary of donor details and experimental workflow employed in this study. FACS-sorted CD3^+^ TCRVδ1^+^ TCRVδ2^−^ thymocytes were analysed at the mRNA level by next-generation sequencing of CDR3 regions of *TRG* and *TRD*. (**a**) Name, age (*d* = days, *m* = months), sex, purity and number of sorted cells of the analysed samples (*n* = 8). (**b**,**c**) Pre- and post-sorting gating strategy. CD3^+^ TCRαβ^−^ TCRVδ1^+^ TCRVδ2^−^ cells were sorted to > 98% final purity. After selection of Singlets (*FSC-A x FSC-W*) and exclusion of debris/dead cells (*FSC-A x SSC-A*), Vδ1^+^ cells were sorted from the CD3^+^αβ^−^ population. (**d**) Amplicons were generated from sorted Vδ1^+^ thymocyte by mRNA/cDNA based multiplex PCR technology. Multiplex primer sets amplify CDR3 regions by targeting Vγ or Vδ and constant gene segments, with the addition of Illumina sequencing adapters as overhangs (red). Sequences were obtained by Illumina MiSeq sequencing and next annotated by IMGT as described in the methods section before downstream bioinformatics analysis. (**e**–**f**) Hierarchical clustering of thymic and PB *TRG* and *TRD* samples using F pairwise similarity metric. Samples were clustered by age (months and days were normalized per year in order to have the same unit of measure - left) and sex (right).

**Fig. 2:**
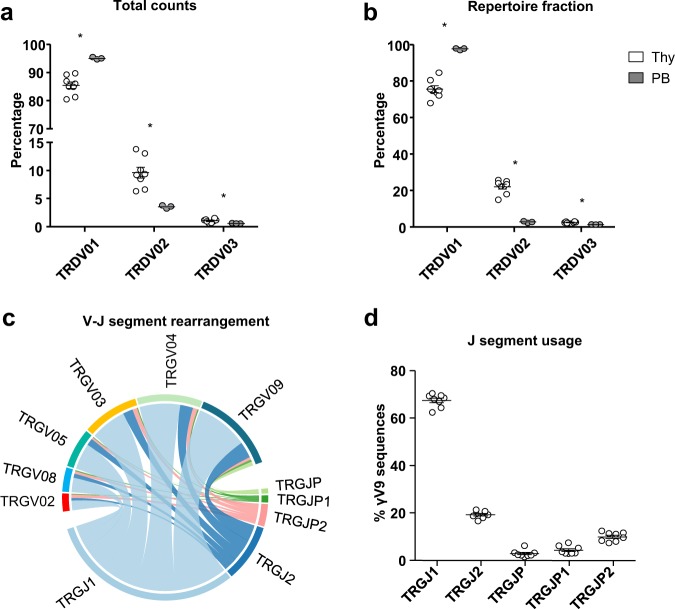
Isolated naïve Vδ1^+^ thymocyte repertoires include a considerable fraction of Vδ2 TCR sequences. (**a**) Total NGS counts and (**b**) relative fraction within repertoire of the thymic TRDV01, TRDV02 and TRDV03 sequences compared to those found in the PB of an unrelated cohort of healthy donors (*n* = 3). Graphical representation of V-J rearrangement in the *TRG* repertoire (**c**) and in the Vγ9 sequences (d) indicates a preferential usage of the J1 segment. Indicated are mean ± SEM; Mann-Whitney test was used to compare groups (*p < 0.05).

In order to study the clonality of the Vδ1^+^ thymocyte population, we collected thymic samples from 8 patients that underwent corrective cardiac surgery (*n* = 8), from 5 days old up to 15 months of age^28^. To ensure the most reliable results and the lowest interferences coming from contaminating cells, we first depleted the thymic samples for αβ T cells and then we sorted CD3^+^ TCRVδ1^+^ TCRVδ2^−^ thymocytes by FACS. Using this purification strategy, we were able to obtain Vδ1^+^ thymocyte samples that were between 98% and 99.6% pure (Fig. 1a–c). Next, we performed NGS experiments on sorted cells as summarized in Fig. 1d. Number of reads and number of unique clonotypes (productive and non-productive) obtained after sequencing of the *TRG* and *TRD* repertoires are listed in Table 2 and Table 3, respectively. Since no clustering for age or sex was observed in the datasets of *TRG* and *TRD* repertoires (Fig. 1e–f), the herein described analyses were made with no subset for these two parameters. Surprisingly, despite the high purities, the NGS analysis revealed that among the retrieved sequences, only around 75% of them were Vδ1 sequences, whereas the remaining fraction was almost entirely constituted of Vδ2 (and much fewer Vδ3) sequences (Fig. 2a,b). For consistency, examples of alignment are supplied as Supplementary Material. Of note, only the chains that were predicted to be functional were considered for the analysis. Importantly, the presence of non-Vδ1 sequences in the pure Vδ1^+^ thymocyte samples was not observed in the PB Vδ1^+^ T cell samples used as controls^33^, where most of the sequences were indeed Vδ1. Thus, the Vδ2 (and Vδ3) sequence pool accounted for around 20% of the whole repertoire of sorted CD3^+^ TCRVδ1^+^ TCRVδ2^−^ thymocytes, whereas it constituted a negligible fraction of the PB samples (Fig. 2b). Importantly, this was true for in every donor analysed (*n* = 8 for thymus, *n* = 3 for PB controls), as highlighted by the minimal standard errors (Fig. 2a,b). Moreover, the analysis of V-J rearrangement in the *TRG* repertoires showed that V segments, including the Vγ9 sequences, rearranged preferentially with the TRGJ1 segment (Fig. 2c,d); whereas they did not include the VγJPVδ2 segment, thus excluding foetal-derived Vγ9Vδ2 T cells.

**Table 2:** Number of reads and number of unique, productive and non-productive clonotypes for the *TRG* repertoires.

Sample (*TRG*)	Reads (#)	Unique clonotypes (#)	Productive clonotypes (#)	Non-productive clonotypes (#)
*Thy01*	124241	76328	67507	8821
*Thy02*	96287	56506	50685	5821
*Thy03*	64503	33011	28952	4059
*Thy04*	79888	45902	40174	5728
*Thy05*	93350	73592	61880	11712
*Thy06*	120272	87512	74731	12781
*Thy07*	67874	53047	46856	6191
*Thy08*	74645	44767	38204	6563
*PB01*	46645	561	561	0
*PB02*	74560	30475	22417	8058
*PB03*	95516	28347	21738	6609

**Table 3:** Number of reads and number of unique, productive and non-productive clonotypes for the *TRD* repertoires.

Sample (*TRD*)	Reads (#)	Unique clonotypes (#)	Productive clonotypes (#)	Non-productive clonotypes (#)
*Thy01*	131535	100266	91681	8585
*Thy02*	121295	87858	80976	6882
*Thy03*	74996	45926	43040	2886
*Thy04*	65350	39273	35977	3296
*Thy05*	183095	158166	142893	15273
*Thy06*	210602	164881	150875	14006
*Thy07*	107236	94185	85930	8255
*Thy08*	118791	70505	63701	6804
*PB01*	58483	744	673	71
*PB02*	158279	39499	32136	7363
*PB03*	126199	41244	34534	6710

**Fig. 3:**
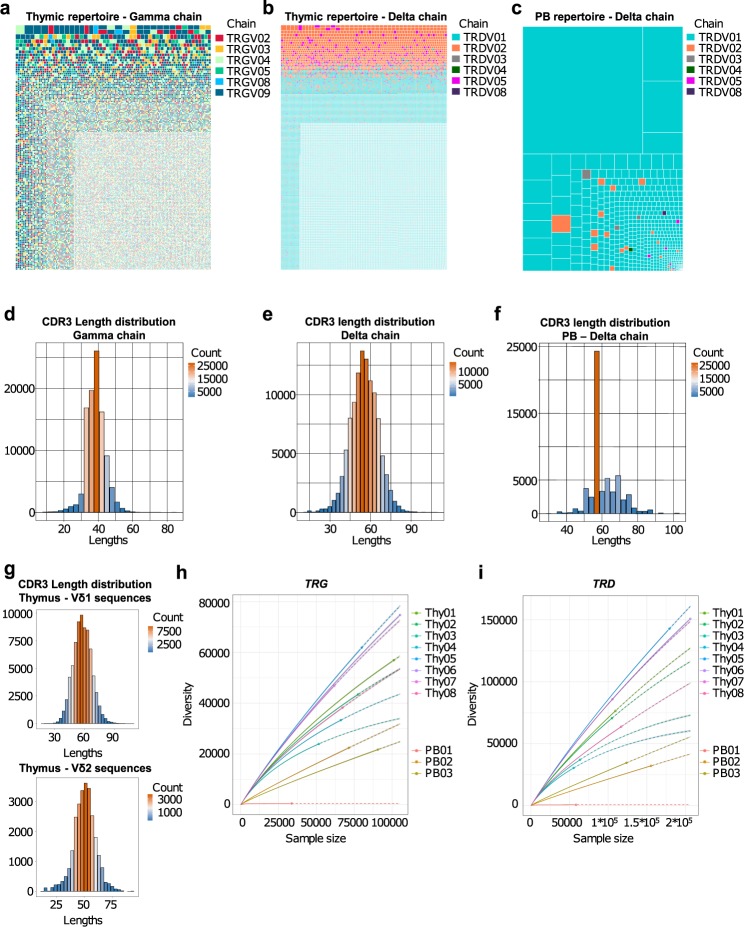
Thymic TCRγ and TCRδ repertoires are highly polyclonal. (**a**,**b**) Graphical representation of the thymic *TRG* (left) and *TRD* (right) repertoires. In these Treemap graphs, each square represents a clonotype bearing a unique nucleotide sequence, its area being proportional to relative abundance in the repertoire; and the colours group the clonotypes by TCR chains. (**c**) Graphical representation of a PB *TRD* control repertoire. (**d**–**e**) Thymic and PB (**f**) CDR3 length (number of nucleotides) distributions for each TCR chain. (**g**) Example of CDR3 length distribution of thymic Vδ1 (top) and Vδ2 (bottom) sequences. (**h**–**i**) Rarefaction analysis of repertoires from thymic and PB samples. The number of unique clonotypes in a sample are plotted against its size. Solid and dashed lines are diversity estimates computed by interpolating and extrapolating using a multinomial model respectively.

We next employed a Treemap type of graph for the clonotype graphical representations (Fig. 3a–c). In these graphs, clonotypes are grouped and colour-coded by chain (TRGV-02, -03, -04, -05, -08 and -09 for the *TRG* datasets; TRDV-01, -02, -03, -04, -05 and -08 for the *TRD* datasets). Other layers of information are included in these graphs. Indeed, each square represents a unique clonotype and its area is proportional the clonotype relative fraction in the dataset. Clonotypes are then ordered by read counts from the most (top left corner) to the least (bottom right corner) abundant. This analysis clearly revealed the presence of many different sequences with very low read counts in the thymic samples, and no skewing towards a specific clonal pattern in either *TRG* or *TRD* repertoires (Fig. 3a,b), whereas repertoires from PB are defined as oligoclonal in the sense that the top 20 clonotypes (by read counts) usually account for more than 70% of the whole repertoire (Fig. 3c). Consistent with this, we found no significant biases in terms of CDR3 length distributions in the thymic samples (Fig. 3d–e), whereas the distribution plot for the PB control shows clear signs of immunological memory with the loss of the typical gaussian-shaped distribution characteristic of naïve repertoires and the acquisition of spikes of more frequent sequences with CDR3 segment of the same length (Fig. 3f). Interestingly, the clonotype graphical representation also highlighted that the non-Vδ1 sequences were the most abundant clonotypes within the repertoire of the purified Vδ1^+^ thymocyte samples (Fig. 3b) and were almost absent in the PB control (Fig. 3c). Also, no differences where observed in the CDR3 distributions of Vδ1 and Vδ2, except (and as expected) for the CDR3 length median value (Fig. 3g).

**Fig. 4:**
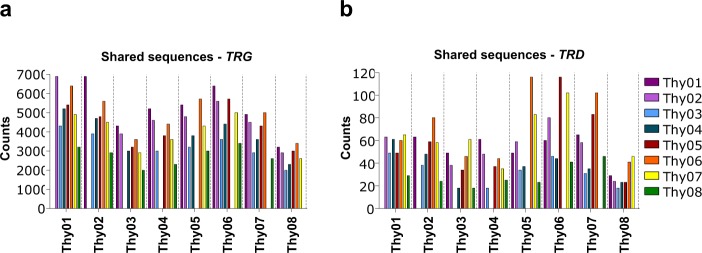
Thymic TCRγ repertoire contains a fraction of public sequences whereas TCRδ is private. (**a**,**b**) Number of shared *TRGV* and *TRDV* sequences across donors (Thy1-8).

Looking more deeply into the clonal diversity of the *TRG* and *TRD* repertoires, we found thymic samples clustering together and separately from the PB controls, with the thymic *TRG* and *TRD* datasets being considerably more diverse than the PB counterparts (Fig. 3h–j). Despite the presence of thousands of low abundant sequences, it was interesting to observe that a considerable number of *TRG* clonotypes was shared between donors, up to 6.9*10^3^ shared sequences between Thy01 and Thy02 samples (Fig. 4a). This phenomenon was clearly restricted to the γ-chain, since the highest number of shared *TRD* clonotypes observed was just 116 between Thy05 and Thy06 samples (Fig. 4b). Thus, whereas common *TRG* sequences are found across thymic Vδ1^+^ samples, their *TRD* repertoires are essentially private.

## Supplementary Information

### ISA-Tab metadata file

Download metadata file.

### Supplementary information

Supplementary Material.

## Data Availability

Custom code used for the processing of the described data can be accessed at figshare^34^.
