# Supplementary material for: High-throughput analysis of the human thymic Vδ1+ T cell receptor repertoire
Source: Sci Data. 2019 Jul 4;6:115. doi: 10.1038/s41597-019-0118-2 (PMC6609709; doi:10.1038/s41597-019-0118-2)
Supplement: Supplementary file 2 — Supplementary Material. [file 41597_2019_118_MOESM2_ESM.pdf]

This supplementary file includes examples of the Vδ1 and Vδ2 alignments obtained with IMGT.

IMGT/V-QUEST program version: 3.4.15; IMGT/V-QUEST reference directory release: 201851-4

Species:	**Homo sapiens**
Receptor type or locus:	**TRD**
IMGT directory reference set:	**F+ORF+ in-frame P**
Search for insertions and deletions:	**no**

Sequence: 1 3

Analysed sequence length:185.

Sequence analysis category: 1 (no indel search)

Sequence compared with the *Homo sapiens* (human) TR set from the IMGT reference directory

>3
tcaagaaagcagcgaaatccgtcgccttaaccatttcagccttgcagctagaagattcag
caaagtacttttgtgctcttgggggccgaccccggcaatccaccggtaaattcgtgtttg
gcacaagttccactgttactcttgtccctgtaaatcagactcgtaacactccttccgttt
ttgtc

**Result summary:**	**Productive TRD rearranged sequence**: (no stop codon and in-frame junction)		
V-GENE and allele	Homsap TRDV1*01 F	score = 356	identity = **98.65%** (73/74 nt)
J-GENE and allele	Homsap TRDJ1*01 F	score = 84	identity = 62.75% (32/51 nt)
D-GENE and allele by IMGT/JunctionAnalysis	Homsap TRDD1*01 F		
FR-IMGT lengths, CDR-IMGT lengths and AA JUNCTION	[X.X.24.11]	[X.X.14]	CALGGRPRQSTGKFVF

V-REGION partial 5' missing nt nb: **199**

Sequence: 2 5

Analysed sequence length:185.

Sequence analysis category: 1 (no indel search)

Sequence compared with the *Homo sapiens* (human) TR set from the IMGT reference directory

>5
tcaagaaagcagcgaaatccgtcgccttaaccatttcagccttacagctagaagattcag
caaagtacttttgtgctcttgggggacggggtccggcgtacaccgataaactcatctttg
gaaaaggaacccgtgtgactgtggaaccaagaagtcagcctcataccaaaccatccgttt
ttgtc

**Result summary:**	**Productive TRD rearranged sequence**: (no stop codon and in-frame junction)		
V-GENE and allele	Homsap TRDV1*01 F	score = 365	identity = **100.00%** (74/74 nt)
J-GENE and allele	Homsap TRDJ1*01 F	score = 255	identity = 100.00% (51/51 nt)
D-GENE and allele by IMGT/JunctionAnalysis	Homsap TRDD3*01 F		
FR-IMGT lengths, CDR-IMGT lengths and AA JUNCTION	[X.X.24.11]	[X.X.14]	CALGGRGPAYTDKLIF

V-REGION partial 5' missing nt nb: **199**

Sequence: 3 9

Analysed sequence length:194.

Sequence analysis category: 1 (no indel search)

Sequence compared with the *Homo sapiens* (human) TR set from the IMGT reference directory

>9
ttaagaaagcagcgaaatccgtcgccttaaccatttcagccttacagctagaagattcag
caaagtacttctgtgctcttggggaactcctactattctactggggatacaccgataaac
tcatctttggaaaaggaacccgtgtgactgtggaaccaagaagtcagcctcataccaaac
cttccgtttttgtc

**Result summary:**	**Productive TRD rearranged sequence**: (no stop codon and in-frame junction)		
V-GENE and allele	Homsap TRDV1*01 F	score = 347	identity = **97.30%** (72/74 nt)
J-GENE and allele	Homsap TRDJ1*01 F	score = 255	identity = 100.00% (51/51 nt)
D-GENE and allele by IMGT/JunctionAnalysis	Homsap TRDD2*01 F and Homsap TRDD3*01 F		
FR-IMGT lengths, CDR-IMGT lengths and AA JUNCTION	[X.X.24.11]	[X.X.17]	CALGELLLFYWGYTDKLIF

V-REGION partial 5' missing nt nb: **199**

Sequence: 4 6

Analysed sequence length:195.

Sequence analysis category: 1 (no indel search)

Sequence compared with the *Homo sapiens* (human) TR set from the IMGT reference directory

>6
tttgcaaagcacctggctgtacttaagatacttgcaccatcagagagagatgaagggtct
tactactgtgcccgtgacactaagccagaagagagggggcctgtgtcctacaccgataaa
ctcatctttggaaaaggaacccgtgtgactgtggaaccaagaaggcagcctcataccaaa
ccatcccgttttgtc

**Result summary:**	**Productive TRD rearranged sequence**: (no stop codon and in-frame junction)		
V-GENE and allele	Homsap TRDV2*01 F, or Homsap TRDV2*02 F or Homsap TRDV2*03 F	score = 322	identity = **97.10%** (67/69 nt)
J-GENE and allele	Homsap TRDJ1*01 F	score = 255	identity = 100.00% (51/51 nt)
D-GENE and allele by IMGT/JunctionAnalysis	Homsap TRDD3*01 F		
FR-IMGT lengths, CDR-IMGT lengths and AA JUNCTION	[X.X.23.11]	[X.X.19]	CARDTKPEERGPVSYTDKLIF

V-REGION partial 5' missing nt nb: **207**

Sequence: 5 10

Analysed sequence length:191.

Sequence analysis category: 1 (no indel search)

Sequence compared with the *Homo sapiens* (human) TR set from the IMGT reference directory

>10
tcaagaaagcagcgaaatccgttgccttaaccatttcagccttacagctagaagattcag
caaagtacttttgtgctcttggggcaattgcccgggggataccttacaccaataaactca
tctttggaaaaggaacccgtgtgactgtggaaccaagaagtcagcctcataccaaaccat
ccgtttttgtc

**Result summary:**	**Productive TRD rearranged sequence**: (no stop codon and in-frame junction)		
V-GENE and allele	Homsap TRDV1*01 F	score = 356	identity = **98.65%** (73/74 nt)
J-GENE and allele	Homsap TRDJ1*01 F	score = 246	identity = 98.04% (50/51 nt)
D-GENE and allele by IMGT/JunctionAnalysis	Homsap TRDD3*01 F		
FR-IMGT lengths, CDR-IMGT lengths and AA JUNCTION	[X.X.24.11]	[X.X.16]	CALGAIARGIPYTNKLIF

V-REGION partial 5' missing nt nb: **199**

Sequence: 6 33

Analysed sequence length:180.

Sequence analysis category: 1 (no indel search)

Sequence compared with the *Homo sapiens* (human) TR set from the IMGT reference directory

>33
attgcaaagaacctggctgtacttaagatacttgcaccatcagagagagatgaagggtct
tactactgtgcctgtgacactggagggggtcggaccaccgataaactcatctttggaaaa
ggaacccgtgtgactgtggaaccaagaagtcagcctcataccaaaccatccgtttttgtc

**Result summary:**	**Productive TRD rearranged sequence**: (no stop codon and in-frame junction)		
V-GENE and allele	Homsap TRDV2*01 F, or Homsap TRDV2*02 F or Homsap TRDV2*03 F	score = 340	identity = **100.00%** (69/69 nt)
J-GENE and allele	Homsap TRDJ1*01 F	score = 246	identity = 98.04% (50/51 nt)
D-GENE and allele by IMGT/JunctionAnalysis	Homsap TRDD3*01 F		
FR-IMGT lengths, CDR-IMGT lengths and AA JUNCTION	[X.X.23.11]	[X.X.14]	CACDTGGGRTTDKLIF

V-REGION partial 5' missing nt nb: **207**

Sequence: 7 46

Analysed sequence length:186.

Sequence analysis category: 1 (no indel search)

Sequence compared with the *Homo sapiens* (human) TR set from the IMGT reference directory

>46
attgcaaagaacctggctgtacttaagatacttgcaccatcagagagagatgaagggtct
tactactgtgcctgtgacacccgtactgggggacaccgaacgtacgataaactcatcttt
ggaaaaggaacccgtgtgactgtggaaccaagaagtcagcctcataccaaaccatccgtt
tttgtc

**Result summary:**	**Productive TRD rearranged sequence**: (no stop codon and in-frame junction)		
V-GENE and allele	Homsap TRDV2*01 F, or Homsap TRDV2*02 F or Homsap TRDV2*03 F	score = 340	identity = **100.00%** (69/69 nt)
J-GENE and allele	Homsap TRDJ1*01 F	score = 219	identity = 92.16% (47/51 nt)
D-GENE and allele by IMGT/JunctionAnalysis	Homsap TRDD3*01 F		
FR-IMGT lengths, CDR-IMGT lengths and AA JUNCTION	[X.X.23.11]	[X.X.16]	CACDTRTGGHRTYDKLIF

V-REGION partial 5' missing nt nb: **207**

Sequence: 8 48

Analysed sequence length:174.

Sequence analysis category: 1 (no indel search)

Sequence compared with the *Homo sapiens* (human) TR set from the IMGT reference directory

>48
attgcaaagaacctggctgtacttaagatacttgcaccatcagagagagatgaagggtct
tactactgtgcctgtgacgcgaaggtgtacaccgataaactcatctttggaaaaggaacc
cgtgtgactgtggaaccaagaagtcagcctcataccaaaccatccgtttttgtc

**Result summary:**	**Productive TRD rearranged sequence**: (no stop codon and in-frame junction)		
V-GENE and allele	Homsap TRDV2*01 F, or Homsap TRDV2*02 F or Homsap TRDV2*03 F	score = 340	identity = **100.00%** (69/69 nt)
J-GENE and allele	Homsap TRDJ1*01 F	score = 255	identity = 100.00% (51/51 nt)
FR-IMGT lengths, CDR-IMGT lengths and AA JUNCTION	[X.X.23.11]	[X.X.12]	CACDAKVYTDKLIF

V-REGION partial 5' missing nt nb: **207**
